# Fibrotic Remodeling during Persistent Atrial Fibrillation: In Silico Investigation of the Role of Calcium for Human Atrial Myofibroblast Electrophysiology

**DOI:** 10.3390/cells10112852

**Published:** 2021-10-22

**Authors:** Jorge Sánchez, Beatriz Trenor, Javier Saiz, Olaf Dössel, Axel Loewe

**Affiliations:** 1 Institute of Biomedical Engineering, Karlsruhe Institute of Technology (KIT), 76131 Karlsruhe, Germany; olaf.Doessel@kit.edu (O.D.); axel.loewe@kit.edu (A.L.); 2 Centro de Investigación e Innovación en Bioingeniería (Ci2B), Universitàt Politècnica de València, 46022 Valencia, Spain; btrenor@eln.upv.es (B.T.); jsaiz@ci2b.upv.es (J.S.)

**Keywords:** myofibroblast, fibrosis, atrial fibrillation, calcium handling

## Abstract

During atrial fibrillation, cardiac tissue undergoes different remodeling processes at different scales from the molecular level to the tissue level. One central player that contributes to both electrical and structural remodeling is the myofibroblast. Based on recent experimental evidence on myofibroblasts’ ability to contract, we extended a biophysical myofibroblast model with Ca^2+^ handling components and studied the effect on cellular and tissue electrophysiology. Using genetic algorithms, we fitted the myofibroblast model parameters to the existing in vitro data. In silico experiments showed that Ca^2+^ currents can explain the experimentally observed variability regarding the myofibroblast resting membrane potential. The presence of an L-type Ca^2+^ current can trigger automaticity in the myofibroblast with a cycle length of 799.9 ms. Myocyte action potentials were prolonged when coupled to myofibroblasts with Ca^2+^ handling machinery. Different spatial myofibroblast distribution patterns increased the vulnerable window to induce arrhythmia from 12 ms in non-fibrotic tissue to 22 ± 2.5 ms and altered the reentry dynamics. Our findings suggest that Ca^2+^ handling can considerably affect myofibroblast electrophysiology and alter the electrical propagation in atrial tissue composed of myocytes coupled with myofibroblasts. These findings can inform experimental validation experiments to further elucidate the role of myofibroblast Ca^2+^ handling in atrial arrhythmogenesis.

## 1. Introduction

Non-myocyte cells are the major population in cardiac tissue (60–70% of cells). In healthy and diseased myocardium, this population is predominantly comprised of cardiac fibroblasts [1]. Once fibroblasts differentiated into myofibroblasts, they are responsible for maintaining the myocardial extracellular matrix homeostasis, which consists mainly of collagen [2,3,4]. Collagen is present in a meager amount in the adult heart [5]. However, due to aging and different pathologies, such as atrial fibrillation (AF), collagen deposit in the cardiac tissue is markedly increased, known as fibrosis [6,7].

Myofibroblast electrophysiology is not clearly described yet; however, several experimental studies have identified protein expression of different ion channels in their membrane (Table 1). Although myofibroblasts are known as non-excitable cells, several studies have shown Na_V_1.5 sodium channel protein expression [8,9,10] and recently also the expression of Ca_V_1 subfamily proteins, which mediate the L-type Ca^2+^ current [9,11,12]. In the last years, there have been a considerable number of studies looking at the role of Ca^2+^ signaling in myo-/fibroblast physiology, and in fibrosis-associated cardiac diseases [13,14,15,16].

Voltage-gated Ca^2+^ channels are an essential part of the cellular electrical machinery, playing a key role in the activation of the sarcoplasmic reticulum channels, contractility, proliferation, and apoptosis of the cell [27,28]. Myofibroblasts exhibit α-smooth muscle actin [29,30] and have been demonstrated to be able to contract [15,31,32], thus indicating a functional Ca^2+^ handling machinery underlying excitation-contraction coupling [12,21,33].

Furthermore, myofibroblasts can be electrically coupled to myocytes via gap junctions and alter the action potential characteristics [28,34,35,36,37]. They can affect the electrical propagation in cardiac tissue during AF or in AF-remodeled tissue and potentially increase arrhythmia propensity [38,39,40]. Key targets of AF upstream therapies are structural changes in the atria, ion channels, and gap junctions [41]. Thus, understanding the role that myofibroblasts play during AF is crucial for improving efficacy of current and future therapies.

Computational models can help understand the mechanisms underlying physical and physiological phenomena at different scales. In silico experiments have the advantage of providing complete control over a wide range of parameters, which in experimental setups are often hard or even impossible to achieve [42]. As a complementary research approach, they can provide insight into the myofibroblast electrophysiology through well-controlled, quantitative experiments that can inform and motivate future in vitro or in vivo studies. Optimization algorithms allow fitting model parameters to in vitro data to study effects on ion channel kinetics [43] and cellular electrophysiology [44,45]. At the tissue level, several studies showed that different patterns and clusters of fibrosis alter the electrical propagation in the cardiac tissue and change the dynamics of reentrant activity [40,46,47].

In this study, we introduce the L-type Ca^2+^ ionic current and a potential intracellular Ca^2+^ handling machinery into the Koivumäki et al. myofibroblast model [10]. Model parameters of the nonlinear system’s ionic channels are fitted to experimental data using a genetic optimization algorithm. We hypothesize that coupling the fitted myofibroblast model with Ca^2+^ handling to an atrial myocyte affects the action potential under electrical remodeling due to persistent atrial fibrillation. We further hypothesize that such changes on the cellular level affect arrhythmia vulnerability at the tissue level when fibrotic infiltration is present.

## 2. Materials and Methods

### 2.1. Myocyte Electrophysiology and Persistent Atrial Fibrillation Remodeling

Human atrial myocyte electrophysiology was represented by the mathematical formulation proposed by Skibsbye et al. [48]. Electrical remodeling due to persistent atrial fibrillation was introduced by changing the maximum conductance of the sodium channel (*g_Na_*), L-type Ca^2+^ channel (*g_CaL_*), transient potassium channel (*g_to_*), ultra-rapid potassium channel (*g_sus_*), slow delayed-rectifier potassium current (*g_Ks_*), rapid delayed-rectifier potassium current (*g_Kr_*), inward potassium rectifier (*g_K_*_1_), Ca^2+^ activated potassium channel (*g_KCa_*), the sodium-calcium exchanger maximum current (*k_NaCa_*), the sarcoplasmic reticulum Ca^2+^ ATPase (SERCA) pump, and ryanodine receptors (RyR), and specific Ca^2+^ handling parameters, such as phospholamban (PLB), sarcolipin (SLN), and the cellular volume as suggested by Skibsbye et al. [48].

### 2.2. Myofibroblast Electrophysiology

**Figure 1:**
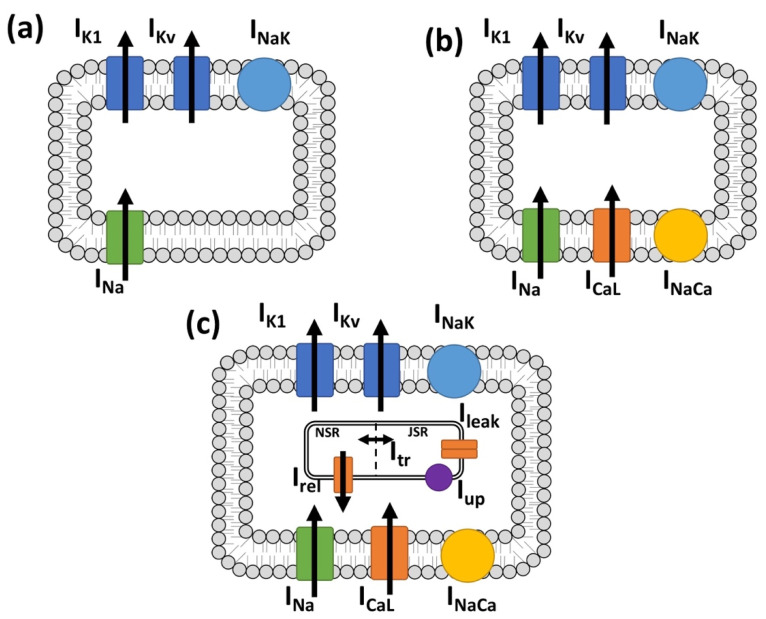
Three different cellular models to explore the effects of myofibroblast Ca^2+^ handling. (**a**) Baseline myofibroblast model without Ca^2+.^ (**b**) Myofibroblast model including the L-type Ca^2+^ current. (**c**) Myofibroblast model including the L-type Ca^2+^ current and an intracellular Ca^2+^ handling system.

The mathematical formulation of fibroblast electrophysiology is based on Koivumäki et al. [10], modified to represent the electrophysiology of a human atrial myofibroblast [40], which in this study is referred to as the baseline myofibroblast model (Figure 1a).

*I_CaL_* parameters (Figure 1b) were fitted to reproduce the experimental data reported by Bae et al. [11]. In the absence of specific experimental data, we assumed that myofibroblasts exhibit a similar phenotype as the myocytes in the regions in which they differentiate. Thus, the intracellular Ca^2+^ handling system was taken from the Courte-manche et al. [49] human atrial myocyte model (Figure 1c). Parameters of the nonlinear. system for Ca^2+^ handling were fitted to reproduce a physiological state that reflects the experimental values for the transmembrane potential and the current traces for *I_Na_* and *I_CaL_* [8,9,10,12,50].

**Figure 2:**
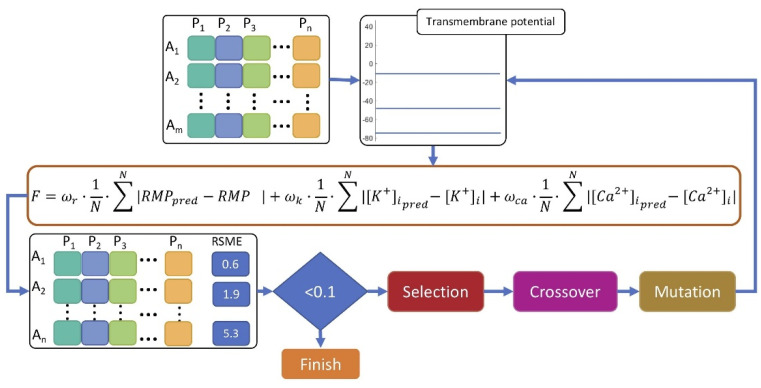
Scheme of the genetic algorithm. Available in vitro data were used as an input to fit the model parameters. The population is comprised of individuals (*A*_1_ to *Am*) with different parameter values representing the “genes” (*P*_1_ to *Pn*, *n*=11). After the transmembrane potential was simulated for all individuals, the fitness function (F) was evaluated. If the error was less than 0.1, the algorithm terminated. Otherwise, it continued by selecting the fittest individuals and allowing crossover and mutation of the genes to generate the next generation population. The process continued until the convergence condition was met.

Figure 2 shows a scheme of the process used to fit the myofibroblast model to the in vitro data. Eleven parameters from the myofibroblast model (“genes” in the genetic optimization algorithm) were assigned random values within the prescribed range to produce the first population. The initial values were constrained to ±5% of their original value in order to constrain the values within a physiological range. Then, single cell. simulations using openCARP [51] were carried out for the entire population to observe the model behavior after 10,000 ms of simulation without stimulation. The value of the transmembrane potential, [*K*^+^]*_i_*, and [*Ca*^2+^]*_i_* were then evaluated according to the fitness function (Equation (1)) under consideration of long term stability of the ionic model by evaluating the last 100 samples (N).

(1) $$F=\omega_{r}\cdot \frac{1}{N}\sum_{}^{N}\left|RMP_{pred}-RMP\right|+\omega_{k}\cdot \frac{1}{N}\sum_{}^{N}\left|{\left[K^{+}\right]}_{i_{pred}}-{\left[K^{+}\right]}_{i}\right|+\omega_{ca}\cdot \frac{1}{N}\sum_{}^{N}\left|{\left[Ca^{2+}\right]}_{i_{pred}}-{\left[Ca^{2+}\right]}_{i}\right|,$$

where *F* is the fitness function, *ω_r_*, *ωk*, and *ω_ca_* are the weighting coefficients, *N* is the number of samples, *RMP* is the transmembrane potential, [*K*^+^]*_i_* is the intracellular potassium concentration, and [*Ca*^2+^]*_i_* is the calcium intracellular concentration.

If the sum of the weighted absolute error was less than 0.1, the algorithm was terminated, providing the parameters of the fittest model. Weights (ωr 1/mV, ωk 1/mMol, and ωca 1/mMol) were the same for transmembrane potential, [K^+^]i, and [Ca^2+^]i. Otherwise, the algorithm continued by selecting the fittest parameter sets and allowing a 0.8 fraction of crossover and mutation rate (following a Gaussian distribution with 0 mean) between the genes of the selected individuals to create the next generation. The process continued until the convergence condition was met.

### 2.3. In Silico Experimental Protocol

In silico experiments of isolated cells were carried out in order to quantify the effect of the electrical coupling between myocytes and myofibroblasts with *I_CaL_* and the Ca^2+^ handling system. Different numbers of myofibroblasts (3, 6, or 9) were coupled to a single myocyte [10,52,53]. The coupling conductance between the myocyte and the myofibroblasts was set to 0.5 nS [38,40]. The myocyte action potential duration at 90 percent repolarization (APD_90_) was measured in an S1–S2 dynamic pacing protocol to obtain the restitution curve.

Tissue simulations were carried in a patch of 50 mm × 50 mm × 3 mm with a central fibrotic area (radius 10 mm) featuring 11 different fibrosis patterns. The patterns were generated as Perlin noise [54], which is used in computer-generated imagery to generate realistic textures [55]. These textures were used to distribute the myofibroblasts following the fibrotic patterns (Figure 5) observed in MRI and histological cuts from interstitial and patchy fibrotic tissue [56,57]. For each pattern, five myofibroblast densities were generated (10%, 20%, 40%, 60%, and 90%). Myofibroblast were assigned to an element and density was computed as the total number of elements in the central area. Thus, 55 different fibrosis patterns were obtained in total to account for the variability of the fibrotic clusters and myofibroblast density distribution. The entropy H of the central fibrotic region was measured as a metric of the fibrosis pattern irregularity:

(2) $$H=-\sum_{k}P_{k}\cdot \log_{2}(P_{k})$$

where *H* is the entropy of the central fibrotic area, *k* is the number of levels (0(myocyte) or 1 (myofibroblast)), and *p* is the probability associated with said binary level.

Tissue conduction velocity was set to 43.39 cm/s [58,59] to represent values reported in patients with persistent atrial fibrillation by adjusting the monodomain conductivity ratio between the longitudinal, transversal, and normal direction in a tissue strand to achieve plane wave conduction velocity. Myofibroblast conductivity was half with respect to the myocyte conductivity [38,40,60].

All meshes had an average edge length of 100 µm to study the electrical dynamics due to the hetero cellular coupling. The tissue was stimulated from one side to simulate a planar wave propagation during sinus rhythm with 10 pulses at a basic cycle length of 1000 ms to reach a limit cycle. Additionally, a second stimulus was introduced to induce an arrhythmia via a cross-field protocol and quantify the tissue’s vulnerable window. The vulnerable window was defined as the total time when a reentry activity was initiated by the cross-field stimulus and maintained for more than 2 s of simulated time. Fibrotic clusters were defined as the areas where all elements of fibrosis were connected. The Euclidean distance between the cluster centroids was measured. Monodomain simulations were performed using a time step of 1 µs to account for the stiff differential equations of the ionic models. All simulations were performed using openCARP. [51].

## 3. Results

### 3.1. Myofibroblast Electrophysiology

**Figure 3:**
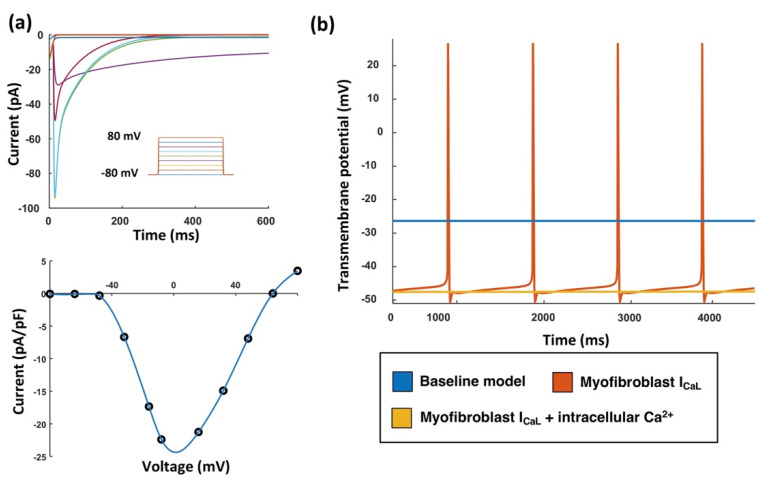
Myofibroblast electrophysiology including the *ICaL* current and the intracellular Ca^2+^ handling system. (**a**) In silico patch-clamp experiment for *ICaL* (top) and comparison (bottom) with in vitro data (dots) from [11]. (**b**) Transmembrane potential of three different non-paced models: baseline model (blue), myofibroblast with *ICaL* (red), and myofibroblast with *ICaL* and intracellular Ca^2+^ handling (yellow).

The genetic algorithm fitted the 11 parameters of the ionic model (Table 2) in order to reproduce experimental results of *I_Na_* [9] and *I_CaL_* [11] (Figure 3a), and the resting membrane potential [2,9,12] (Figure 3b). The resulting set of parameters (Table 2) represents a model that reached a steady state with no stimulation. Long-term stability was tested using two different approaches, quiescent steady-state without stimulating the myofibroblast and reaching a limit cycle when stimulating the cell with a train of 100 pulses at a basic cycle length of 1000 ms.

An in silico patch-clamp experiment was conducted in order to measure the maxi mal current at different potentials. The fitted *I_CaL_* current reproduced the results from Bae et al. [11], as depicted in Figure 3a. The transmembrane voltage course without pacing for the three myofibroblast models is depicted in Figure 3b. When *I_CaL_* was added to the baseline myofibroblast model, the myofibroblast exhibited a maximum diastolic potential of –34 mV. This potential was not a stable resting membrane potential, but the addition of the *I_CaL_* current to the baseline model made the myofibroblast exhibit spontaneous activity with a cycle length of 799.9 ms. The diastolic depolarization rate measured over the first 100 ms time interval (DDR_100_) was 62.60 mV/s. When in addition to *I_CaL_* an intracellular Ca^2+^ handling system was added, the automaticity of the cell ceased and a stable resting membrane potential of –46 mV was reached.

**Figure 4:**
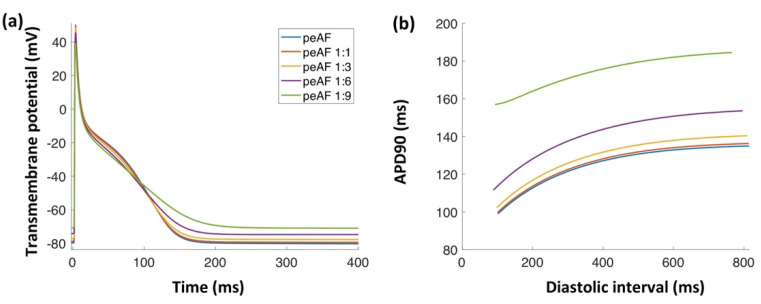
Fibroblast coupling effect on the myocyte action potential. (**a**) Uncoupled AF-remodeled myocyte action potential and four different numbers of myofibroblasts coupled to a single myocyte. (**b**) Action potential restitution curves for AF-remodeled myocytes and four different numbers of myofibroblasts coupled to a single myocyte.

When a single myocyte was coupled to different numbers of myofibroblasts (including *I_CaL_* and the intracellular Ca^2+^ handling system), the myocyte action potential duration was affected. Control myocyte APD_90_ was shortened from 180 ms and when coupling to 3, 6, or 9 myofibroblasts, 110 ms, 70 ms, and 50 ms, respectively. Myocyte APD_90_ with AF electrical remodeling was 130 ms. When coupled to 3, 6, or 9 myofibrob-lasts, it was prolonged to 163 ms, 168 ms, and 185 ms, respectively (Figure 4a). Myocyte action potential amplitude was reduced from 129.09 mV (non-coupled) to 127.84 mV, 124.94 mV, 118.91 mV, and 109.14 mV for 1, 3, 6, and 9 coupled myofibroblasts, respectively. Additionally, the action potential maximal upstroke change (dV/dt_max_), for control myocytes, was increased from 119.3 mV/s (non-coupled) to 120.1 mV/s when coupled to 9 myofibroblasts. AF-remodeled myocyte dV/dt_max_ was slightly reduced from 111.5 mV/s (non-coupled) to 110.9 mV/s when coupled to 9 myofibroblasts.

The myocyte resting membrane potential was depolarized (from –74 mV to –60 mV) when coupled to 9 myofibroblasts. Additionally, APD90 restitution curve maximum slope was increased for 1, 3, and 6 coupled myofibroblasts (3.59, 3.69, and 3.79, respectively) in comparison to the slope of the uncoupled AF-remodeled myocyte (2.97). However, the slope was flattened when coupled to 9 myofibroblasts (slope of 1.83) (Figure 4b).

### 3.2. Tissue Simulations

**Figure 5:**
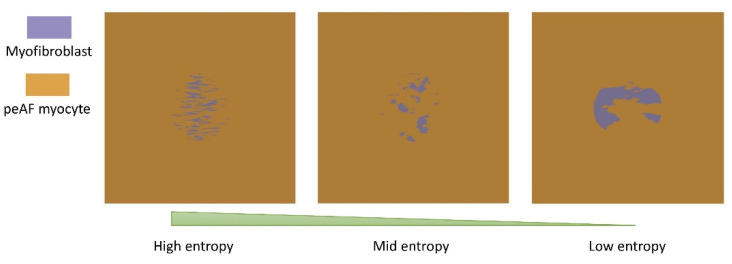
Three different fibrosis patterns with clustering of the myofibroblast infiltration in the central part of the tissue for a density of 20%. From left to right, patterns that resemble interstitial to patchy fibrosis.

Figure 5 depicts three different patterns with three different entropy values. Patterns of myofibroblast infiltration that resemble interstitial fibrosis had a higher entropy compared to the patterns of myofibroblast infiltration that resemble patchy fibrosis. The elongated patterns resembling interstitial fibrosis with a considerable number of gaps in the central area of the tissue are more complex, which increases the entropy value. Clusters of patchy fibrosis have a more regular distribution of myofibroblasts, giving lower entropy values.

**Figure 6:**
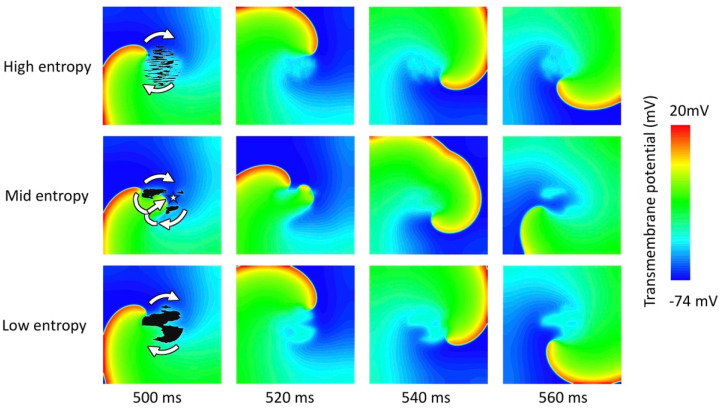
Snapshots of the transmembrane potential at different time instances for three different fibrosis patterns with 20% myofibroblast density (black areas in the left column represent myofibroblasts). White arrows indicate the reentry trajectory, and the white star shows the exit point of the reentry. Reentrant activity was initiated by a cross-field stimulus. The top row shows the reentry around the fibrotic region of high entropy that resembles interstitial fibrosis. The middle row shows the reentrant activity with an exit point due to the separation of the clusters with a mean entropy value that resembles disperse patchy fibrosis. The bottom row shows reentrant activity around an area of low entropy that resembles a pattern of patchy fibrosis.

Tissue-level simulations revealed that myofibroblasts change reentry dynamics depending on their density and pattern. Fibrosis patterns with high entropy (interstitial fibrosis) increased the tissue vulnerability to reentrant activity (Figure 6 first row). Tissue vulnerability was increased due to the slow conductive fibrotic area, which acts as a conduction block when interstitial fibrosis has a density higher than 40%. Patterns with mean entropy values (resembling a transition from interstitial fibrosis to patchy fibrosis) stabilize reentrant activity. Due to the distance between fibrotic clusters (mean distance 1.2 ± 0.2 mm), the non-fibrotic islands can act as exit points that appear as focal activity. Patchy fibrosis with low entropy did not markedly exhibit exit points for the reentrant activity (Figure 6 last row) but stabilized the reentrant activity around the fibrotic area.

**Figure 7:**
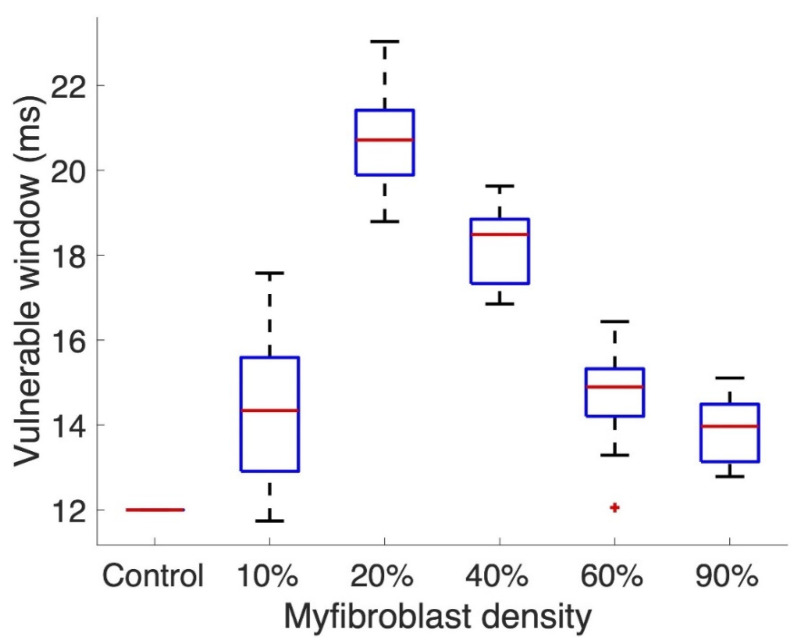
Tissue vulnerable window for all myofibroblast infiltration patterns. Vulnerability to arrhythmia increased for all patterns. Outliers are marked with a plus (+) on the boxplot.

Tissue vulnerability mainly depended on the myofibroblast density independent from the infiltration pattern. Increasing myofibroblast density increased the vulnerable window (Figure 7): from 12 ms (non-fibrotic tissue) to 14 ± 1.78 ms, 20 ± 1.13 ms, 18 ± 0.92 ms, 15 ± 1.28 ms, and 14 ± 0.83 ms for 10%, 20%, 40%, 60%, and 90%, respectively.

## 4. Discussion

In this work, we have introduced a human atrial myofibroblast model, which includes the L-type Ca^2+^ and an intracellular Ca^2+^ handling system. This model is based on in vitro data and assumptions following Occam’s razor when no specific data were available. In silico experiments using this new model can help to explore and potentially understand the electrophysiology of the human atrial myofibroblast. We showed that intracellular calcium handling can tip the scales of cellular automaticity and that myofibroblast infiltration patterns can promote and maintain reentrant activity.

Our fitting methodology yielded a myofibroblast model with long-term stability that is able to reproduce patch-clamp experiment recordings from in vitro data. Despite the lack of data, the model yielded physiological transmembrane potential courses in accordance with different experimental works [9,61].

Ca^2+^ signaling has been described as a pathway of fibroblast proliferation and differentiation [62]. In vitro and in vivo studies showed that the number of myofibroblasts and collagen in rat hearts were reduced by blocking L/T-type Ca^2+^ channels [63,64,65]. These studies suggest that Ca^2+^ ion channels and intracellular Ca^2+^ handling plays an essential role in the electrophysiology of myofibroblasts and the development of cardiac fibrosis. Nevertheless, the specific effect of voltage-gated Ca^2+^ channels and their impact on myofibroblast electrophysiology has not yet been fully described. In our study, using in silico experiments and based on the notion that myofibroblasts can contract, we were able to study the effect of the expression of L-type Ca^2+^ voltage-channel and the intracellular Ca^2+^ handling system on myofibroblast electrophysiology. We observed two different behaviors: first, the presence of L-type Ca^2+^ current triggers automatic activity in isolated myofibroblasts. Second, the presence of the intracellular Ca^2+^ handling system, which was composed of the sarcoplasmic reticulum and the Ca^2+^ SERCA pump, stops this automaticity and leads to a steady-state with a resting membrane potential of –46 mV, which is in accordance with experimentally reported values [9,38,53,61]. Human sinus node cells, which also exhibit automaticity, showed a diastolic depolarization rate (DDR100) of around 76 mV/s and a cycle length of around 828 ms [66,67,68]. The myofibroblast model with only the L-type Ca^2+^ channel had a DDR100 of 62.60 mV/s and a cycle length of 799.9 ms, which could potentially trigger ectopic activity [69]. Automaticity in the myofibroblast model was driven by a membrane clock formed by incomplete inactivation of the ICaL current. This mechanism is different from a funny current-driven membrane clock observed in pacemaker cells as part of a coupled clock [70]. In cardiac pacemaker cells, automaticity is driven by the interplay of membrane and calcium clock in contrast [71]. The observed automaticity is a result of the in vitro data used in the fitting algorithm which show no complete ICaL inactivation at hyperpolarized membrane voltages. Complete inactivation would likely stop automaticity but would not be in line with the available in vitro data. The addition of L-type Ca^2+^ current and intracellular Ca^2+^ handling system to the myofibroblast model showed a different effect when coupled to a single myocyte. A previous study [40] showed that the myofibroblast baseline model shortens the APD90. Our results showed a prolongation of the APD90 when increasing the number of coupled myofibroblasts. Nattel et al. [72] reviewed the importance of Ca^2+^ for the function of the atrial cardiomyocytes and its relation to AF. In this study, we present how Ca^2+^ can alter the electrophysiology of the human atrial myofibroblast. In particular, myofibroblast automaticity was only observed in the absence of Ca^2+^ handling machinery. While the relevance of myofibroblast automaticity for atrial ectopy is unclear [50], abnormal disrupted Ca^2+^ handling could be a contributor to arrhythmia triggers and help to understand arrhythmogenicity and may hold potential for future therapeutic approaches [14].

Tissue simulations showed that not only could the myofibroblast infiltration affect the arrhythmia vulnerability [73,74], but also the fibrosis pattern can play a role in the initiation and maintenance of reentrant activity [46,47,54,75]. Sridhar et al. [76] have shown the role that fibroblast infiltration plays regarding the onset of arrhythmia due to the increase of anisotropy in the tissue. In another study, Kazbanov et al. [77] showed that diffuse fibrosis patterns promote the onset of arrhythmia. The fitted myofibroblast model has altered the tissue window vulnerability compared to a previous study [40] where the vulnerable window was similar for different densities of myofibroblasts. The observed mechanism underlying the widening of the vulnerable window in our simulations was a slowing of conduction. Additionally, a shortening of the vulnerable window was observed when the tissue became less excitable due to high densities of myofibroblasts, which led to a block in conduction as previously observed [40]. In combination, our results show a biphasic behavior of the tissue vulnerable window as observed by other studies [40,78]. It is worth noting that when the myofibroblast density increases, the vulnerability of tissue is decreased due to conduction blocks created by high density clusters of myofibroblasts.

Our results show that patterns that resemble low degree patchy fibrosis with low entropy did not markedly affect the tissue vulnerability [79]. However, patterns with mean entropy values that correspond to a cluster of fibrosis with a separation distance between centroids of 1.2 ± 0.2 mm did promote reentrant activity and exhibit exit points that could be identified as focal points [80]. In addition, when entropy values increased and fibrosis was interstitial, the reentrant activity was maintained around the fibrotic area. Maintenance of reentry around interstitial fibrosis is due to the depolarization of the central area which slows down conduction [73] and does not create zig-zag propagation paths observed in fibrosis with mainly collagen [46].

Based on reports that myofibroblasts can contract [15,31,32] and the hypothesis that they express a similar phenotype as the myocytes in the region in which they differentiate [81,82,83], we developed an extended myofibroblast model. The extended myofibroblast model explores one possible way of Ca^2+^ entry via the L-type Ca^2+^ channel to the intracellular medium and the intracellular Ca^2+^ handling. Nevertheless, Ca^2+^ signaling in the myo-/fibroblast has been shown to be mostly mediated by TRP channels. Different studies suggest that under the presence of TGF-β1, myofibroblast TRPM7 channels are activated and contribute to Ca^2+^ influx [13,26,84]. Du et al. [26] have shown that TRPM7 channels are important for the differentiation of fibroblasts into myofibroblasts and play a role in fibrogenesis in human AF. TRPM7 channels are activated by a decrease in the free Mg^2+^ concentration. However, the role of low Mg^2+^ in patients with AF has not yet been clarified [84,85,86]. In this study, we considered the L-type Ca^2+^ current as the primary contributor to Ca^2+^ influx and did not consider any changes in the Mg^2+^ concentration. Another possible Ca^2+^ entry could be via CRAC channels (Orai/STIM), which are essential regulators of intracellular Ca^2+^ homeostasis in different cells of the cardiovascular system [87]. Camacho-Londoño et al. [88] reported that Orai channels Ca^2+^ entry may be mediated via the activation of angiotensin II-induced signaling cascades in cardiac fibroblasts but have not reported the effect on the myofibroblast electrophysiology or the effect on electrical propagation. These findings are a motivation to further study the influence of Ca^2+^ in the differentiation of fibroblasts in the cardiac tissue and their importance for cardiac arrhythmias. The scarcity of experimental data is the main limitation of this work, rendering independent validation of the myofibroblast electrophysiology impossible at the current stage. As such, this study should be considered hypothesis-generating, aims to stimulate discussion in the scientific community, and hopes to inform future experimental work.

We did not consider paracrine effects [12,14,79], which are likely present during the inflammatory process as one of the pathways that triggers fibroblast differentiation to myofibroblasts or the effect of collagen that might affect dynamics of the reentrant activity [52,89]. In addition, it will be of great interest to study how the ratio of electrically coupled fibroblasts and myofibroblasts in cardiac tissue can alter the dynamics of atrial fibrillation [90]. Moreover, the proposed model in this study could potentially be used as a basis to study the mechano-electrical properties of myofibroblasts and their effect on the atrial tissue during AF. Additionally, the Courtemanche et al. [49] mathematical formulation of intracellular calcium handling was used due to the smaller number of parameters compared with the Koivumäki et al. [91] mathematical formulation of intracellular calcium handling that would increase the level of uncertainty due to the higher number of parameters which are still under study in the myofibroblast electrophysiology. Quantitative data to inform the choice of their values are mostly lacking. Moreover, we did not consider the influence of the atrial anatomy, which might change the dynamics of the reentrant activity [92,93].

## 5. Conclusions

In silico models can integrate the available in vitro data on myofibroblast elec- trophysiology and provide additional insight based on them. Ca^2+^ handling in the myofibroblast alters the cellular electrophysiology and prolongs the action potential when coupled to an atrial myocyte. If only the L-Type Ca^2+^ membrane channel is added, myofibroblasts exhibit sustained automaticity. Tissue simulations show that myofibroblast infiltration increases the vulnerability of the tissue to arrhythmia and that different fibrosis patterns change the dynamics of the reentrant activity. Future experiments based on these findings will further elucidate the role of calcium in the myofibroblast and the electromechanical effects of coupling between myofibroblasts and myocytes.

## Figures and Tables

**Table 1:** Reported cardiac myo-/fibroblast ion channels from different locations and species.

Type of Ion Channel	Ion Channel/Protein	Cell Types
Voltage-gated Na^+^	Nav1.2; Nav1.5; Nav1.9	Human ventricular fibroblast [17]; human atrial fibroblast [8,9,10]
Voltage-gated K^+^	Kv4.1, Kv4.2, Kv4.3, Kv6.2	Human ventricular fibrob last [17]; human atrial fibrob last [9]
K^+^ inward rectifier	Kir2.1	Human ventricular fibroblast [17]; dog ventricular fibroblast [18]
Voltage-gated Ca^2+^	Cav1.2, Cav1.3	Human ventricular fibroblast [11]; human atrial fibroblast [9]
ATP-activated K^+^	SUR2/Kir6.1	Mice ventricular fibroblast [19]; rat ventricular fibroblast [20]
Ca^2+-^activated K^+^	KCa1.1, KCa3.1	Human ventricular fibroblast [17]; human atrial fibroblast [21]
Voltage-Gated Cl^−^	ClCN3	Human ventricular fibroblast [17]
Store-operated Ca^2+^ or Receptor-operated Ca^2+^	Orai1/STIM1	Rat atrial fibroblast [16]; Human ventricular fibroblast [22]; Rat ventricular fibroblast [23]
TRP	TRPC3 [24] TRPV4 [25] TRPM1 [9] TRPM7 [12,26] TRPA1 [12]	Rat ventricular fibroblast [24,25]; Human atrial fibrob last [9,12,26]

**Table 2:** Myofibroblast model parameters including *ICaL* with the intracellular Ca^2+^ handling system ionic conductance modifications.

*gKv* (nS/pF)	*gK*1 (nS/pF)	*gNa* (nS/pF)	*gNab* (nS/pF)	*gNaK* (nS/pF)	*gCaL* (nS/pF)	*gCab* (nS/pF)	*kNaCa* (nS/pF)	*kpCa* (nS/pF)	*rKv* (nS/pF)	*sKv* (nS/pF)
1.34	1.79	0.73	2.39	0.84	0.48	0.57	2.55	1.0	14.04	16.21

## Data Availability

The model files used in the simulations with openCARP (https://www.opencarp.org) and presented in this study are openly available at https://github.com/jorge221/myofibroblast_characterization. and in the CellML repository.
